# Systematic narrative review of modalities in physiotherapy for managing pain in hip and knee osteoarthritis: A review

**DOI:** 10.1097/MD.0000000000038225

**Published:** 2024-09-27

**Authors:** Jean-Philippe Paul Berteau

**Affiliations:** a Department of Physical Therapy, City University of New York—College of Staten Island, New York City, NY; b New York Center for Biomedical Engineering, City University of New York—City College of New York, New York City, NY; c Nanoscience Initiative, Advanced Science Research Center, City University of New York, New York City, NY.

**Keywords:** electrical stimulation, heat therapy, osteoarthritis, ultrasound

## Abstract

Osteoarthritis (OA) affects 528 million individuals globally, predominantly in knee and hip joints, with a notable impact on females aged over 55, resulting in a substantial economic burden. However, the efficacy of modalities used in physiotherapy to manage OA pain for reducing the need for joint replacement remains an open question, and guidelines differ. Our systematic narrative review, drawing from reputable databases (e.g., PubMed, Cochrane, and CINAHL) with specific Mesh terms investigated evidence from 23 Randomized Controlled Trials (that included a control or a sham group in 30 different protocols) using therapeutic modalities like ultrasound, diathermy, and electrical stimulation for knee and hip OA pain, involving a total of 1055 subjects. We investigated the attainment of minimal clinically important differences in pain reduction, operationalized through a 20% decrement in the Western Ontario and McMaster University Arthritis Index or Visual Analog Scale (VAS) score. Our results indicated that 15 protocols out of 30 reach that level, but there were no statistical differences among modalities. Half of the protocol presented in the literature reached clinical efficiency but studies on hip remains scarce. We recommend a comprehensive, sequential, and multimodal intervention plan for individuals with joint OA with initial transcutaneous electrical nerve stimulation and progressing to a 2-week protocol of continuous ultrasound, potentially combined with deep microwave diathermy. Long-term intervention involves the use of pulsed electrical stimulation. For hip OA, a cautious approach and discussions with healthcare providers about potential benefits of spinal cord nerve stimulation.

## 1. Introduction

The global prevalence of osteoarthritis (OA) reached 528 million people in 2019,^[1]^ reflecting a notable 113% increase since 1990 with an annual economic burden estimated at $136.8 billion, exclusive to the United States. A significant majority, 73%, of those affected are aged over 55, and 60% are female, with the knee being the most commonly affected joint, followed by the hip and hand.^[1–3]^ Despite the growing use of joint replacement,^[4]^ it is considered a costly last resort. Thus, the management of OA emerges as a significant challenge in orthopedics, impacting approximately 344 million individuals with moderate or severe severity levels,^[3]^ hinting at potential benefits from rehabilitation. Here, we propose a thorough exploration of the intricate pathogenesis of musculoskeletal disorders to offer guidelines for the nonpharmaceutical evidence-based strategies currently used in physiotherapy and to effectively alleviate OA pain and decrease the need for joint replacement. We focused on modalities that do not need the active participation of the patients to be clinically applicable to the highest number of people living with OA.

**Figure 1.**
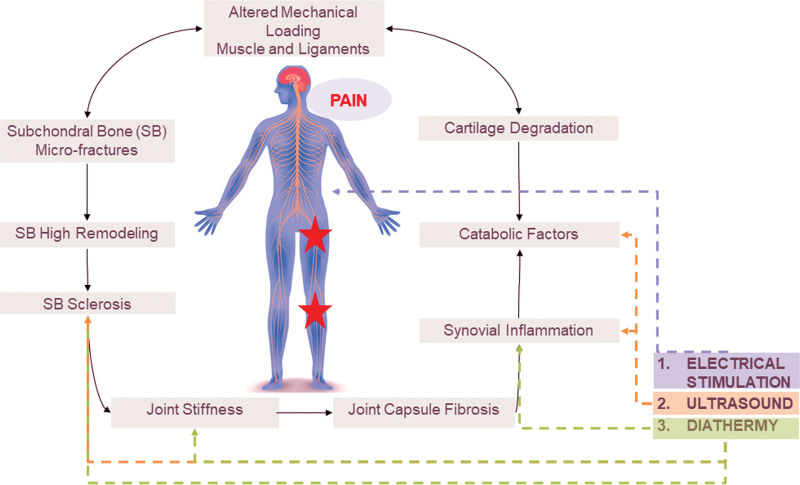
Pathophysiological progression of OA vicious cycle and the impact of pain management modalities. Ultrasound modulates reduces inflammation, stimulates tissue regeneration, and alter catabolic factor. Diathermy raises tissue temperature which improves tissue elasticity, and decrease inflammation. ES interventions, such as TENS, NMES, IFC, and others, engage (i) homotopical inhibition via large nerve fibers and (ii) descending pain inhibition systems. ES = electrical stimulation, IFC = interferential current, NMES = neuromuscular electrical stimulation, OA = osteoarthritis, TENS = transcutaneous electrical nerve stimulation.

OA evolves through the progressive degradation of articular cartilage and subchondral bone (SB) microfractures, and understanding its development hinges on the dynamic interplay between cartilage and SB^[5–7]^ as depicted in Figure 1. In the initial stages, OA goes beyond mere cartilage degradation solely, resulting from abnormal loading that induces shear forces on it; there is also altered SB remodeling that impacts catabolic factors and acts as a critical trigger.^[8–11]^ Regarding the cartilage, compositional, and structural alterations in chondrocytes, such as hypertrophy due to aging or oxidative stress, set the stage for the production of catabolic factors, including cytokines (e.g., interleukin-6, interleukin-8), chemokines (e.g., regulated on activation, normal T cell expressed, and secreted, interferon gamma-induced protein 10), metalloproteases (matrix metalloproteinase 1, matrix metalloproteinase 3), and heat-shock proteins (e.g., heat-shock protein family A member 1A). As the pathological process unfolds, triggered by the production of catabolic factors, the articular cartilage undergoes degradation, rendering it incapable of fully absorbing physiological and physical forces. Thus, the cartilage, usually effective at preventing biomechanical damage under normal conditions, fails to repair adequately and gets destroyed, increasing mechanical forces on the SB tissue. SB in early OA stages presents high remodeling and low mineralization from the gradual increase in mechanical forces that increase the bone remodeling process rate. At a later stage, the increase in mechanical forces triggers the genetic production of enhancers of mineralization by SB osteoblasts through the mechanic-transduction^[12,13]^ that thickens and hardens the tissue, a process called bone sclerosis, which ends up with the formation of bone cysts and marginal osteophytes.^[14,15]^ In addition, simultaneous alterations of both articular cartilage and SB are associated with synovial inflammation and joint capsule fibrosis, causing the loss of range of motion, stiffness, tenderness, and pain, intensifying the impact throughout the joint.^[16]^ The self-sustaining vicious cycle of OA unfolds, with each stage influencing and amplifying others and creating the intricate landscape of OA pathogenesis^[5,6]^ (Fig. 1). Thus, joint alterations,^[17–19]^ such as the narrowing of the joint space and a brighter SB, contribute to key OA features seen on imaging after a patient complains of pain (e.g., X-ray, dual X-ray absorptiometry, computerized tomography). Regardless of the biomarkers used to track OA progression, pain is the first clinical sign that alerts patients to initiate OA treatment. Its reduction is one of the primary outcomes of clinical intervention.

In the context of OA-related pain, intricate peripheral and central mechanisms come into play. Nerve sensitizations, integral to pain transmission in OA patients, contribute to the complex interplay between pain and joint pathology. While hyaline cartilage is not innervated, pain arises from the synovium, SB, and periosteum, all innervated by small-diameter nociceptive neurons. Indeed, the stimuli generated by tissue damage during joint degradation result in pain, with structural factors such as bone marrow lesions, synovial thickening (synovitis), and knee effusion associated with it. The inflammatory mediators produced by the synovium and chondrocytes intensify the excitation of nociceptive neurons, creating an amplified painful response. While pharmaceutical interventions, exercise, and bracing therapy have demonstrated efficacy^[2,3,6,16,20–29]^ in alleviating pain for individuals with OA affecting either the knee or hip joints, available evidence regarding modalities used in physiotherapy remains scarce, namely heat therapy,^[30]^ ultrasound therapy,^[31]^ and electrical stimulation (ES),^[32]^ as depicted in Figure 1, for proposing clear, practical guidelines to enhance clinical outcomes and mitigate of OA-related pain.

We conducted a systematic narrative review of scholarly literature from reputable databases (e.g., Pubmed, Cochrane, and CINAHL) to address this issue. The review employed specific Mesh terms and synonyms related to OA, physiotherapy, and pain. The overarching aim was to distill evidence from randomized control trials, emphasizing the attainment of minimal clinically important differences (MCID) in pain reduction, operationalized through a 20% decrement in the Western Ontario and McMaster University Arthritis Index (WOMAC) or Visual Analog Scale (VAS) score.

## 2. Methods

In our dedicated exploration of the current state of treating OA, we employed a state-of-the-art narrative review as our chosen methodology. Recognizing the efficacy of pharmaceutical interventions, exercise, and bracing therapy in pain alleviation for OA patients, we focused on physiotherapeutic approaches. Our comprehensive evaluation sourced scholarly literature from reputable databases such as Pubmed, Cochrane, and CINAHL. To ensure methodological transparency, we utilized specific Mesh terms and their synonyms related to OA, physiotherapy, and pain. While not delving into the exhaustive details characteristic of a systematic review, we complied with the Scale for the Assessment of Narrative Review Articles which assesses aspects such as the article’s significance, formulation of questions, literature search description, referencing, scientific reasoning, and data presentation. The primary objective was to distill evidence from randomized control trials, emphasizing the achievement of MCID in pain reduction for conservative management^[33,34]^ which was operationalized through a 20% decrement in either the WOMAC or VAS score. In addition to our systematic narrative review of the scholarly literature, we analyzed the data acquired to explore statistical significance between different treatment groups and assess the relationship with the duration of treatment, using parametric or nonparametric according to the distribution of the data evaluated with the Shapiro–Wilk test. This analytical phase was conducted employing International Business Machines Corporation Statistical Package for the Social Sciences Statistics (Version 27).

## 3. Observations and discussions

**Figure 2.**
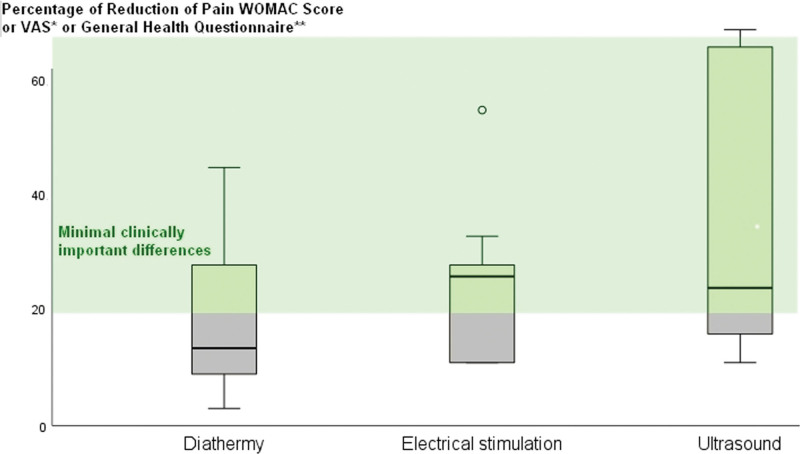
Duration versus percentage of pain reduction in ultrasound studies for knee and hip osteoarthritis. The x-axis represents treatment duration in weeks, while the y-axis signifies the percentage of pain reduction. Studies exceeding the minimum clinical efficiency threshold (set at 20%, based on literature evidence) are enclosed within a highlighted frame for clearer identification and comparison.

We examined various therapeutic modalities for managing knee and hip OA pain from 23 Randomized Controlled Trials (that included a control or a sham group in 30 different protocols) using therapeutic modalities like ultrasound, diathermy, and ES for knee and hip OA pain (Table 1). Ten protocols with a combined participant pool of 354 individuals were identified within the ultrasound category. The heat therapy section included 7 protocols, with 262 participants for knee-related and 155 participants for hip-related interventions. For ES, 9 protocols were reviewed, covering 249 participants for knee-related treatments and 35 participants for hip-related treatments. Across all modalities, the cumulative participation in these diverse interventions reached 1055 individuals. Analyzing the mean percentage of pain reduction based on the WOMAC Score, VAS, or General Health Questionnaire for each category, no statistical differences were observed among the groups, as illustrated in Figure 2. Furthermore, there was no statistical relationship between the percentage of pain reduction and the duration of treatment.

**Table 1:** Comparative analysis of knee and hip osteoarthritis interventions encompassing US, heat therapy, and ES modalities, including CU, PU, H-PSWD, and TENS.

Authors	Interventions	n (group) = sample size	Treatment duration	Percentage of reduction of pain WOMAC Score or VAS* or General Health Questionnaire**
Ultrasound		Knee		
Boyaci et al^[35]^	Continuous CU	n (CU) = 33	2 wk	16
Alfredo et al (2020)	Group mixed US method	n (CU) = 100	2 wk	25
Özgönenel et al (2008)	Group (CU)	n (CU) = 34	2 wk	18
Luksurapan and Boonhong^[36]^	Group (CU) (prepost)	n (CU) = 23	2 wk	69
Loyola-Sánchez et al (2012)	Group pulsed ultrasound (PU)	n (PU) = 14	8 wk	23
Kozanoglu et al^[37]^	Group (CU) (prepost)	n (CU) = 30	2 wk	66
Külcü et al (2009)	Group (CU)	n (CU) = 15	3 wk	44
Karakaş et al (2020)	Group (PU)	n (CU) = 39	8 wk	11
		HIP		
Köybaşi et al^[38]^	Group (CU)	n (CU) = 15	2 wk	66*
Király et al (2022)	Group (PU)	n (CU) = 18	2 wk	11.31*
Heat		Knee		
Laufer et al (2005)	H-PSWD	n (H-PSWD) = 32	3 wk	9
Laufer et al (2005)	L-PSWD	n (L-PSWD) = 38	3 wk	3
Laufer et al (2005)	S-SWD	n (S-SWD) = 33	3 wk	11
Giombini et al^[39]^	MD	n (MD) = 30	4 wk	45
Giombini et al^[39]^	S-MD	n (S-MD) = 25	4 wk	3
Rattanachaiyanont and Kuptniratsaikul^[40]^	SWD	n (SWD) = 50	3 wk	28
Rattanachaiyanont and Kuptniratsaikul^[40]^	S-SWD	n (S-SWD) = 54	3 wk	28
		HIP		
Fioravanti et al^[41]^	Phyto thermotherapy (PHY)*	n (PHY) = 109	2 wk	40*
Kovács et al (2016)	BLX	n (BLX) = 20	3 wk	13
Moffet et al (1996)	PSW therapy**	n (PSW) = 26	3 wk	14**
Electrical stimulation		Knee		
Atamaz et al^[42]^	TENS	n (TENS) = 29	3 wk	11
Atamaz et al^[42]^	IFC	n (IFC) = 27	3 wk	11
Pietrosimone et al^[43]^	TENS + therapeutic Exercise	n (TENS) = 30	4 wk	11
Adedoyin et al^[44]^	TE + ES	n (TENS) = 16	4 wk	27
Garland et al^[45]^	Pulse ES	n (ES) = 38	12 wk	26
Fary et al^[46]^	Pulse ES	n (ES) = 34	26 wk	11
Shimoura et al (2019)	TENS stair climb	n (TENS) = 25	0	33*
Shimoura et al (2019)	TENS timed up and go	n (TENS) = 25	0	26*
Shimoura et al (2019)	TENS 6 min-walk test	n (TENS) = 25	0	55*
		HIP		
Cottingham et al^[47]^	SCNS	n (SCNS) = 35	2 wk	28*

Sample sizes, treatment durations, and pain reduction percentages (measured via WOMAC Score, VAS*, or General Health Questionnaire**) are detailed across the studies.

BLX = balneotherapy and exercise, CU = continuous ultrasound, ES = electrical stimulation, H-PSWD = high-pulse short wave diathermy, IFC = interferential current, L-PSWD = low-pulse short wave diathermy, MD = microwave diathermy, PSW = pulsed short wave, PU = pulsed ultrasound, SCNS = spinal cord nerve stimulation, S-MD = sham microwave diathermy, S-SWD = sham short wave diathermy, SWD = short wave diathermy, TENS = transcutaneous electrical nerve stimulation, VAS = Visual Analog Scale, WOMAC = Western Ontario and McMaster University Arthritis Index.

### 3.1. Heat therapy

Within the realm of heat therapy, diathermy stands as a prominent modality extensively investigated for addressing hip and knee pain associated with primary OA. The underlying principle of diathermy lies in its capacity to increase the temperature of underlying tissues. Recent evidence^[48]^ shows that heat therapy’s physiological effects encompass pain relief, heightened blood flow, increased metabolism, and enhanced elasticity of connective tissue. TRP vanilloid 1 receptors, which are ion channels activated by noxious heat, mediate neural transduction of heat in primary afferent neurons, the spinal cord, and throughout the brain. The activation of TRP vanilloid 1 receptors in the brain has the potential to modulate antinociceptive descending pathways. Two commonly employed forms of diathermy are shortwave diathermy (SWD) and microwave diathermy (MD). SWD utilizes high-frequency electromagnetic energy to generate pulsed or continuous wave heat. At the same time, MD employs microwaves to generate heat on superficial tissues, with lower-frequency waves that do not penetrate deep muscles. The mechanism of action for MD involves increasing local blood flow and facilitating the delivery of nutrients and oxygen to promote tissue repair. It enhances capillary permeability, allowing macrophages and granulocytes to remove toxins and necrotic debris, interferes with inflammatory process enzymes, and induces the expression of heat-shock proteins crucial for proper protein folding and waste removal.

**Figure 3.**
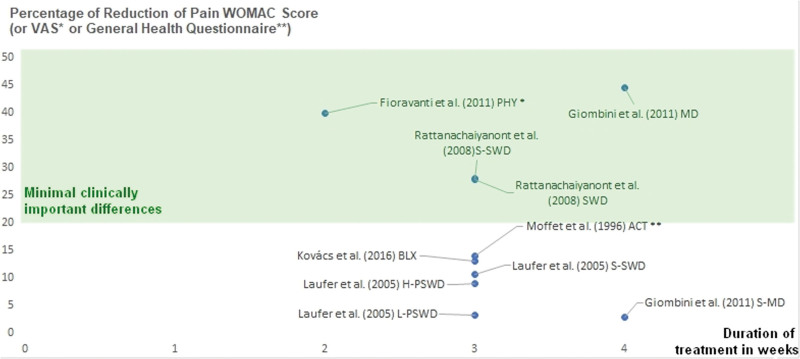
Duration versus percentage of pain reduction in heat studies (diathermy) for knee and hip osteoarthritis. The x-axis represents treatment duration in weeks, while the y-axis signifies the percentage of pain reduction. Studies exceeding the minimum clinical efficiency threshold (set at 20%, based on literature evidence) are enclosed within a highlighted frame for clearer identification and comparison.

Table 1 synthesizes findings from diverse studies exploring the impact of diathermy interventions on knee and hip pain, visually represented in Figure 3. In Figure 3, the x-axis corresponds to treatment duration in weeks, and the y-axis represents the percentage of pain reduction. Within the highlighted section, 3 studies surpass the established minimum clinical efficiency threshold of 20%, whereas 6 studies fall below this benchmark. Among the effective interventions, Giombini et al^[39]^ investigated MD and sham MD (S-MD), reporting a 44.6% reduction with MD and 2.9% with S-MD. Rattanachaiyanont and Kuptniratsaikul^[40]^ examined SWD and sham SWD (S-SWD), revealing a 27.9% and 28.1% reduction in pain, respectively. Since there was a minimal amount of study related to hip pain dealing with diathermy, we included the one of Fioravanti et al,^[41]^ who explored Phyto Thermotherapy (PHY), indicating a 40.0% reduction, supporting the efficiency of heat at the hip level as well. In relation to this, our results on diathermy support the recent findings of a systematic review with a meta-analysis of 21 studies,^[49]^ focusing on WOMAC Index and VAS pain, which highlights significant enhancements in both functional scores and relief from painful symptoms post mud-bath therapy, heat therapy performed in a spa, for knee OA.

Consequently, deep MD, operating at 434 MHz for 30 minutes 5 times a week, demonstrated efficacy in reducing synovial thickness, a prognostic marker of cartilage loss. This intervention proved most effective, showcasing an 8% to 45% decrease in WOMAC scores for various diathermy treatments. Notably, both diathermy and sham therapy improved WOMAC scores, with no significant difference observed between SWD and sham diathermy. The conclusions highlight deep MD as an effective intervention for knee OA.

### 3.2. Ultrasound therapy

Ultrasound therapy has emerged as a promising therapeutic avenue for effectively managing pain associated with hip and knee OA. Its fundamental principle involves converting electrical energy into heat as it traverses tissues, with similar effects of diathermy (see above), influencing neuromuscular activity for muscle relaxation and promoting tissue regeneration while concurrently reducing inflammation. For instance, pro-inflammatory cytokines, such as interleukin-1β and tumor necrosis factor-α, play a role in joint pain, and ultrasound has been reported to decrease their levels in vitro and vivo.^[50]^ In addition, recent evidence^[51]^ depicts that continuous mode ultrasound, often employed in chronic pain conditions, induces muscle relaxation and increases blood flow, fostering tissue regeneration and reducing inflammation. Pulsed ultrasound (PU), chosen for acute and subacute injuries, minimizes thermal impact while preserving other biological effects, such as acoustic cavitation and increased cell permeability. To summarize, ultrasound’s main biological effects stem from its absorption of mechanical energy and subsequent heat production in tissues that induces an anti-inflammatory response.

**Figure 4.**
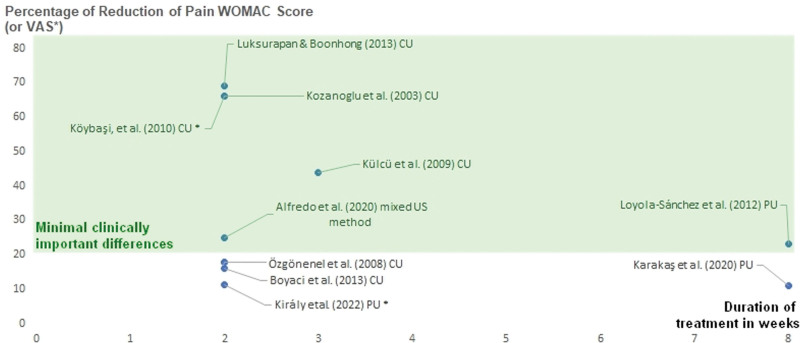
Duration versus percentage of pain reduction in electrical stimulation studies for knee and hip osteoarthritis. The x-axis represents treatment duration in weeks, while the y-axis signifies the percentage of pain reduction. Studies exceeding the minimum clinical efficiency threshold (set at 20%, based on literature evidence) are enclosed within a highlighted frame for clearer identification and comparison.

Table 1 consolidates findings from various studies exploring the impact of ultrasound interventions on knee and hip pain, as depicted in Figure 4. The x-axis of Figure 4 represents treatment duration in weeks, while the y-axis indicates the percentage of pain reduction. Six studies surpassing the minimum clinical efficiency threshold (set at 20%, based on literature evidence) are enclosed within a highlighted frame, while 4 are not. In knee-related studies, continuous ultrasound (CU) and mixed ultrasound methods exhibit substantial pain reductions ranging from 16% to an impressive 69% over 2 to 8 weeks. PU interventions also display noteworthy decreases ranging from 11% to 23% over 8-week durations. Analyzing the outcomes of Luksurapan and Boonhong and Kozanoglu et al,^[36,37]^ utilizing CU interventions for knee OA, reveals a consistent trend toward pain reduction. Luksurapan and Boonhong’s study,^[36]^ employing a 2-week CU intervention with a prepost design and a sample size of 23 participants, demonstrates an impressive 69% reduction in pain.

In contrast, Kozanoglu et al’s investigation,^[37]^ with a larger sample size of 30 participants, reports a substantial 66% reduction in pain over the same duration. These findings suggest that a 2-week protocol involving CU may yield significant pain reduction benefits for individuals with knee OA. For instance, the parameters used in both studies with the highest efficiency were continuous mode, 1 MHz frequency and 1 W/cm^2^ conducted 5 times a week for 2 weeks for a total of 10 sessions. With only the duration of application differed (5–10 minutes).

In the context of hip pain, studies utilizing CU and PU, report reductions of 66% and 11.31% over 2-week periods, indicating a potential role for ultrasound interventions in hip pain management. However, the challenge lies in attributing observed pain relief solely to ultrasound therapy, given its frequent integration with other modalities. Here, we did not include a study related to extracorporeal shock wave therapy since, according to a recent systematic review,^[52]^ there is low to very low-quality evidence suggesting that extracorporeal shock wave therapy may provide clinical improvements in pain compared to a sham intervention considering the MCID. On the contrary, another recent systematic review with meta-analysis about using phonophoresis,^[53]^ which employs ultrasound to deliver therapeutic drugs through skin absorption, has shown promise in alleviating pain and inflammation in OA pathology. Still, it’s dependent on the medications used.

### 3.3. Electrical stimulation

ES has emerged as a promising intervention for tackling muscle weakness and alleviating knee and hip OA symptoms. Employing various modalities such as high-frequency transcutaneous electrical nerve stimulation, low-frequency transcutaneous electrical nerve stimulation, neuromuscular electrical stimulation (NMES), interferential current (IFC), pulsed ES (PES), and noninvasive interactive neurostimulation, these interventions aim to target the muscles and mitigate the impact of OA symptoms. Pain relief through ES in OA is linked to the gate-control theory, wherein non-nociceptor fibers are stimulated to reduce pain sensation by inhibiting pain-transmitting fibers.^[32]^ Neuromuscular ES also stimulates the release of endogenous analgesics, providing indirect pain relief by reducing muscle spasms, increasing strength, and preventing muscle degeneration associated with chronic myofascial pain. According to recent evidence,^[54]^ one of the prevailing pain relief methods, transcutaneous electrical nerve stimulation (TENS), operates through diverse mechanisms. High-frequency, low-intensity TENS triggers homotopical inhibition via large nerve fibers. In contrast, low-frequency, high-intensity TENS activates descending pain inhibition systems, fostering connectivity between the sensorimotor cortex and prefrontal regions. This intricate interplay influences ongoing brain activity modulates alpha oscillations, and facilitates top-down inhibitory control, collectively underpinning the analgesic efficacy of TENS. Regarding PES,^[45]^ it demonstrates promising biological effects on articular cartilage, as evidenced by increased gene transcription promoting the synthesis of collagen components and the suppression of destructive enzymes and inflammatory mediators, analogous to bone stimulator therapy based on mechanotransduction.

**Figure 5.**
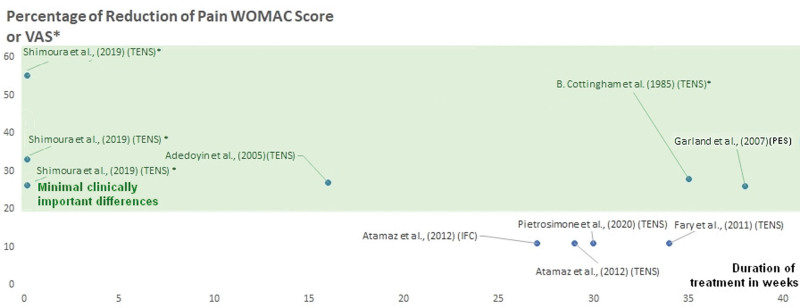
Comparative analysis of diathermy (heat), electrical stimulation, and ultrasound modalities in knee and hip osteoarthritis. Box plots with standard error bars depict mean and standard deviation for each modality, revealing no statistically significant differences. Studies surpassing the minimum clinical efficiency threshold (set at 20%, based on literature evidence) are emphasized within a highlighted frame, facilitating identification and comparison of efficacious interventions.

Table 1 consolidates findings from diverse studies investigating the impact of ES interventions on knee and hip pain, as illustrated in Figure 5. The x-axis in Figure 5 denotes treatment duration in weeks, while the y-axis signifies the percentage of pain reduction. Within the highlighted frame, 6 studies exceed the minimum clinical efficiency threshold of 20%, while 4 do not. Noteworthy reductions in pain above the 20% threshold were observed in pain management strategies utilizing TENS, both independently and in combination with therapeutic exercise or PES. TENS interventions exhibited significant efficacy in alleviating pain at rest and during functional tests, showcasing substantial reductions in pain during activities such as stair climb, timed up and go, and a 6-minute walk test, but as an immediate relief for the patient. The parameters used in most of the studies using TENS here stayed in the range of the last guidelines: frequency between 50 and 75 Hz with a pulse duration between 200 and 400 microseconds and a treatment duration of 20 minutes.^[32]^ In addition, results from Adedoyin et al,^[44]^ show the best results by combining TENS with exercise. Thus, our findings align with a recent systematic review^[55]^ of 29 studies (involving 1398 individuals with knee OA), which showed that active TENS significantly relieves pain, improves function, and enhances walking ability and that combining with exercise demonstrated superior outcomes, especially in the medium and long term.^[55]^ For long-term outcomes, our results show that PES has a long-term benefits, as demonstrated by a 3 months randomized controlled trial^[45]^ using a knee garment with flexible, embedded electrodes and a small battery-operated generator that produced a 100-Hz, negative pulsed signal. In addition to this, in another recent systematic review^[56]^ of 6 randomized controlled trials examining the addition of NMES to exercise programs for knee OA, the evidence regarding its effectiveness, particularly in pain management, still needs to be conclusive. Thus our findings support the use of TENS as a first intention and then PES as a long-term intervention.

Regarding data on hip pain, interventions were more limited, with only spinal cord nerve stimulation (SCNS) by Cottingham et al^[47]^ showing a significant 28% reduction over 2 weeks. While these findings underscore the potential benefits of various interventions for OA pain, additional research is necessary to evaluate their long-term efficacy and compare their effectiveness, particularly concerning hip pain. According to our findings, no studies have explored IFC and hip OA pain. IFC’s potential for delivering a more substantial pain relief effect with its alternating electrical current penetrating deeper presents a promising avenue for future exploration in hip OA treatment. Considering the anatomical differences between the knee and hip, a deeper electrical current may prove more effective in future studies of ES and hip OA, opening new possibilities for improved pain management strategies.

### 3.4. Comparative analysis

Our comprehensive review integrates findings from various studies that investigate the impact of diathermy, ultrasound, and ES interventions on knee and hip pain. In the highlighted sections of Figures 3–5, a collective total of 15 studies (3, 6, and 6, respectively) surpass the established minimum clinical efficiency threshold of 20%, while 16 studies (6, 4, and 6, respectively) fall below this benchmark. Noteworthy effective interventions include MD, SWD, CU, mixed ultrasound methods, and TENS, both independently and in combination with therapeutic exercise.

Based on this review, we can propose the following clinical practice guidelines for people living with OA. For an individual with knee OA, a comprehensive intervention protocol may include a combination of evidence-based therapies. Begin with a regimen of CU or mixed ultrasound methods, as demonstrated by studies such as Boyaci et al^[35]^ and Luksurapan and Boonhong,^[36]^ showcasing substantial pain reductions ranging from 16% to an impressive 69% over treatment periods of 2 to 8 weeks. The recommendation is 1 MHz frequency and 1 W/cm^2^ conducted 5 times a week for 2 weeks for a total of 10 sessions between 5 and 10 minutes. Incorporate TENS, supported by Atamaz et al^[42]^ and Pietrosimone et al,^[43]^ exhibited an 11% reduction in pain over 3 to 4 weeks. Complement these interventions with therapeutic exercises tailored to strengthen the muscles around the knee, as recommended by Fary et al^[46]^ Additionally, considering the positive outcomes of ES modalities in knee pain management, the inclusion of NMES or IFC can be discussed with the healthcare provider, as hinted by the data provided. For an individual grappling with hip OA, the intervention protocol should be approached cautiously due to the limited data available. Begin with CU or PU interventions, as evidenced by Köybaşi et al^[38]^ and Király et al,^[57]^ reporting reductions of 66% and 11.31% over 2-week periods. Further research is needed to consider the challenges in establishing clinical efficiency for specific interventions like pulsed shortwave therapy and the potential benefits of SCNS and NMES.

Regarding published systematic review with meta-analysis on other modalities,^[58,59]^ both pulsed electromagnetic field therapy and extracorporeal shock wave therapy (ESWT) demonstrated effectiveness in treating OA of the knee. Pulsed electromagnetic field therapy, as indicated by a meta-analysis of 16 studies,^[58]^ exhibits clinically significant effects on pain, stiffness, and physical function in OA patients compared to a placebo. Similarly, ESWT proves effective for knee OA,^[59]^ providing immediate pain relief within 2 weeks, with sustained effects up to 1 year. ESWT’s positive histological impacts include enhanced SB anabolism and improved trabecular microarchitecture. Additionally, it influences biochemical factors, reducing inflammatory cytokines and normalizing cytokine levels in osteoarthritic chondrocytes. While both therapies show promising short-term and long-term outcomes, none of the systematic reviews evaluated their clinical improvement.

Our review focused on measures of clinical improvement (minimum clinically important difference), using the consensus method, which was 20% of the maximum score for pain from WOMAC and VAS.^[60]^ Still, other studies’ data suggest that minimum clinically important difference may be variable (2.2–27.6 out of 100) depending on the outcome measurement and original pain.^[61]^ Therefore, the blanket application of 20% may not be suggested regardless of the tool used, so it will depend on the clinical practitioner to evaluate the most appropriate level for their patient. While these metrics assess meaningful clinical effects, recent discussions have highlighted their limitations by not accounting for costs, risks, benefits, and treatment inconvenience. The concept of the smallest worthwhile effect,^[62]^ determined through the benefit-harm trade-off method, identifies the minimal improvement perceived as worthwhile by patients, considering benefits outweighing risks and inconvenience, and is compared against natural recovery. Other studies explored the “patient acceptable symptom state”,^[63]^ representing the symptom state deemed acceptable or when patients feel “well” after treatment. Patient-acceptable symptom state estimates for WOMAC function exceeded the minimum important change median estimate of 17, suggesting varied perspectives on clinical improvement. While the minimal clinically important change score remains commonly used, the evolving research in this field holds promise for incorporating value judgments alongside clinical considerations in interpreting intervention trials. Although we have diligently presented each subscale in the results section, we recognize the importance of exploring a subgroup review based on the type of WOMAC or VAS. Nevertheless, a notable challenge arises from the limited number of publications, hindering the potential for a comprehensive and robust subgroup analysis due to insufficient data for exhaustive exploration.

Clinical practice guidelines for joint OA may propose a comprehensive, multimodal intervention plan with a sequential approach. Initially, TENS is recommended, utilizing a frequency between 50 and 75 Hz, a pulse duration of 200 to 400 microseconds, and a treatment duration of 20 minutes. Subsequently, a 2-week protocol introduces CU, incorporating parameters such as continuous mode, 1 MHz frequency, and 1 W/cm^2^, administered 5 times a week for a total of 10 sessions. This regimen may be complemented by deep MD operating at 434 MHz for 30 minutes, 5 times a week. For long-term intervention, PES is suggested. In the case of hip OA, a cautious approach is advised, focusing on CU, PU, and potential interventions like SCNS and NMES. Regular follow-ups and personalized treatment plans, considering individual variations and preferences, are deemed crucial. Further research is necessary to advance the understanding of interventions, especially for hip OA, and establish standardized protocols for optimal pain management.

## 4. Conclusion

This comprehensive review of interventions for hip and knee OA underscores the intricate landscape of therapeutic modalities. The key takeaway from the presented findings is the recommendation for a comprehensive, sequential, and multimodal intervention plan for individuals with joint OA. Initiating with TENS and progressing to a 2-week protocol of CU, potentially combined with deep MD, sets the foundation for effective pain management. Long-term intervention involves the use of PES. A cautious approach for hip OA, focusing on various modalities including CU, PU, SCNS, and NMES, is advised. Personalized treatment plans, regular follow-ups, and the need for further research to establish standardized protocols emphasize the importance of tailoring interventions to individual needs for optimal pain relief in joint OA.

## Author contributions

**Conceptualization, Data curation, Formal analysis, Methodology, Project administration, Software, Supervision, Writing—original draft, Writing—review & editing:** Jean-Philippe Paul Berteau.
